# Comprehensive Proteomic Profiling Reveals Dysregulation of Angiogenesis and Inflammatory Pathways in the Brains of SIRT3 Knockout Mice

**DOI:** 10.3390/brainsci16030270

**Published:** 2026-02-28

**Authors:** Qingping He, Samia Khan, Linlin Wang, Gordon C. Ibeanu, P. Andy Li

**Affiliations:** Department of Pharmaceutical Sciences, Biomanufacturing Research Institute Technology Enterprise (BRITE), College of Health and Sciences, North Carolina Central University, Durham, NC 27707, USA; qhe@nccu.edu (Q.H.); samiaahammedkhan@gmail.com (S.K.); lwang4@nccu.edu (L.W.); gibeanu@nccu.edu (G.C.I.)

**Keywords:** angiogenesis, apoptosis, brain, chemokine, cytokine, mouse, proteomics, sirtuin 3

## Abstract

Background: Sirtuin 3 (SIRT3), a mitochondrial NAD^+^-dependent deacetylase, plays a central role in regulating mitochondrial metabolism, oxidative stress, and cell survival. Although SIRT3 has been implicated in angiogenesis, apoptosis, and inflammation, its global proteomic impact on the brain remains unclear. This study aimed to systematically characterize alterations in angiogenesis-, apoptosis-, chemokine-, and cytokine-related proteins in the brains of SIRT3 knockout (SIRT3 KO aka SIRT3^−^/^−^) mice compared with wild-type (WT) controls. Methods: Adult male C57BL/6 WT and SIRT3 KO mice were analyzed using proteome profiler antibody microarrays covering 53 angiogenesis factors, 21 apoptosis markers, 28 chemokines, and 111 cytokines. Protein expression changes were quantified by chemiluminescence imaging and densitometric analysis. Results: The results showed a distinct suppression of angiogenic proteins (amphiregulin, angiogenin, DPPIV, GM-CSF, IGFBP-2, IGFBP-3, IL-1β, PDGF-AA, PDGF-BB, proliferin, serpin F1, thrombospeondin-2, TIMP-4, and VEGF-B), activation of both pro-apoptotic (BAD, cytochrome *c*, Smac/DIABLO, HIF-1α, Fas, TNF R1, and TRAILR2) and anti-apoptotic, stress-related proteins (Bcl-x, catalase, HO/HMOX2, HSP27, HSP70, and MCL1) in the SIRT3 KO animals compared with the WT controls. Notably, SIRT3 deficiency was associated with increased expression of inflammatory mediators linked to glial activation and neurodegeneration (BLC/CCL13, LIX/CXCL5, MIG/CXCL9, chitinase 3-like 1, CCL22/MDC, IL-6, myeloperoxidase, osteopontin, RBP4, Reg3G, and TNF-α), alongside disturbed proteins involved in immune surveillance and vascular remodeling (6Ckine/CCL21, chemerin, DF, EGF, fractalkine/CX3CL1, HGF, IGFBP-6, IL-16, and I-TAC). Conclusions: Collectively, these findings demonstrate that SIRT3 is a key regulator of mitochondrial-dependent vascular, apoptotic, and neuroimmune pathways in the brain, and that its loss creates a molecular environment consistent with heightened vulnerability to neurodegenerative processes.

## 1. Introduction

Sirtuins are a family of nicotinamide adenine dinucleotide (NAD^+^)-dependent class III histone deacetylases that regulate cellular metabolism, mitochondrial biogenesis and function, free radical production, and energy production. Seven mammalian sirtuin homologs (SIRT1–SIRT7) have been identified, among which SIRT1 and SIRT3 are the most extensively studied. SIRT3 is predominantly localized in mitochondria, although its presence in the nucleus and cytosol has also been reported [1]. The physiological functions of SIRT3 include promoting mitochondrial biogenesis via deacetylation of PGC-1α, inducing mitophagy through deacetylation of PINK1/Parkin, regulating mitochondrial dynamics via FOXO3, and attenuating oxidative stress by suppressing ROS formation and upregulating antioxidant systems such as SOD2, glutathione, and thioredoxin [2]. In addition, SIRT3 modulates several fundamental cellular processes, including angiogenesis, apoptosis, chemokine production, and inflammatory signaling.

Angiogenesis, the formation of new blood vessels from pre-existing ones, plays a critical role in tissue growth and development. Under pathological conditions, however, angiogenesis could be either detrimental or beneficial. For example, while angiogenesis supports tumor growth and metastasis, it can also facilitate post-stroke repair and promote neurological recovery [3,4,5,6]. Consequently, the modulation of angiogenesis represents a key therapeutic target for numerous diseases. Loss of SIRT3 impairs angiogenic capacity, whereas SIRT3 overexpression in cardiac microvascular endothelial cells restores vessel sprouting and tube formation [7].

Apoptosis, a programmed form of cell death triggered by a cascade of biochemical reactions, is essential for embryogenesis, tissue homeostasis, and development. Aberrant regulation of apoptosis—either suppression or overactivation—can contribute to diverse pathological states. In the nervous system, neurodegenerative diseases, ischemic stroke, and traumatic brain injury are associated with the activation of apoptotic pathways. The extrinsic cell death pathway is initiated by inflammatory cytokines binding to their receptors, which subsequently activates caspase-8 and downstream cascades. The intrinsic cell death pathway is mediated through mitochondria. A critical event in the intrinsic pathway is the formation of the mitochondrial permeability transition pore (mPTP), a large protein channel that enables the release of pro-apoptotic factors, such as cytochrome c, Smac/DIABLO, apoptosis-inducing factor (AIF), and endonuclease G (EndoG). These proteins activate cytoplasmic caspases or translocate to the nucleus to induce DNA fragmentation [8]. The mPTP is formed through the interaction of mitochondrial F_1_F_0_-ATP synthase, the adenine nucleotide translocator (ANT), and matrix cyclophilin D (CypD). Upon activation via acetylation, CypD translocates from the mitochondrial matrix to the inner membrane, where it binds ANT and F-ATP synthase to assemble the mPTP [9]. SIRT3 inhibits mPTP formation by deacetylating and inactivating CypD, thereby preventing mitochondrial-mediated apoptosis [10,11,12].

Chemokines are a specialized group of small cytokines whose primary function is to direct the migration (chemotaxis) of immune cells—including neutrophils, monocytes, lymphocytes, and eosinophils—to sites of infection, inflammation, or injury. During infection or tissue damage, resident cells, such as macrophages, fibroblasts, and endothelial cells, release chemokines to recruit circulating immune cells, thereby coordinating inflammatory responses and promoting tissue repair. Depending on the tissue type and pathological context, SIRT3 has been shown to either suppress or enhance chemokine production [13,14,15].

Inflammation serves as a crucial defense mechanism for the body, responding to harmful stimuli like pathogens, toxins, or damaged cells. However, when inflammation becomes uncontrolled, it can lead to various diseases. Its primary goals are to eliminate harmful agents, remove damaged tissue, and initiate repair. However, chronic or dysregulated inflammatory signaling underlies many acute and chronic disorders, including autoimmune, metabolic, cardiovascular, and neurodegenerative diseases. In the central nervous system (CNS), neuroinflammation plays a pivotal role in the initiation and progression of conditions such as Alzheimer’s disease, Parkinson’s disease, multiple sclerosis, amyotrophic lateral sclerosis, Huntington’s disease, stroke, traumatic brain injury, and epilepsy [16,17,18,19,20,21,22]. SIRT3 is a key regulator of inflammatory processes; overexpression of SIRT3 has been shown to suppress macrophage activation by reducing the production of pro-inflammatory cytokines and signaling mediators such as TNF-α, NF-κB, and the NLRP3 inflammasome [23]. Although most studies highlight the anti-inflammatory properties of SIRT3, there is evidence that indicates context-dependent pro-inflammatory effects. For example, Guan et al. reported that SIRT3-mediated deacetylation of NLRC4 enhances inflammasome activation and promotes inflammatory signaling [24].

Angiogenesis, apoptosis, chemokine signaling, and inflammation are not independent biological processes, but are tightly interconnected components of the cellular stress response, particularly within metabolically active tissues such as the brain. These signaling networks converge at the level of mitochondrial function, redox balance, and metabolic adaptation—processes that are centrally regulated by SIRT3. Mitochondrial ROS production and bioenergetic status influence endothelial cell survival and angiogenic capacity, dictate the activation of intrinsic apoptotic pathways, and shape inflammatory and chemokine signaling through redox-sensitive transcription factors and inflammasome activation. Conversely, inflammatory cytokines and chemokines can impair mitochondrial function and promote apoptotic signaling, creating a feed-forward cycle of tissue injury and repair. By regulating mitochondrial protein acetylation, oxidative phosphorylation efficiency, antioxidant defenses, and mitochondrial permeability transition, SIRT3 occupies a nodal position linking vascular remodeling, cell survival, immune cell recruitment, and inflammatory signaling. Therefore, the simultaneous profiling of angiogenesis-, apoptosis-, chemokine-, and inflammation-related proteins will provide a biologically integrated framework to assess how SIRT3 deletion reshapes mitochondrial-driven signaling networks in the brain.

To date, no study has systematically characterized the protein expression profiles of angiogenic factors, apoptotic mediators, chemokines, and inflammatory cytokines in SIRT3 knockout (SIRT3 KO) mice. Aside from a single transcriptomic study reporting gene expression change in the liver of SIRT3 KO mice [25], little is known about how SIRT3 deletion alters the proteomic landscape in the brain. Therefore, this study aims to investigate differences in the expression of angiogenesis-, apoptosis-, chemokine-, and inflammation-related proteins between the SIRT3 KO and WT mouse brain using a proteomic microarray approach.

## 2. Materials and Methods

### 2.1. Animals and Tissue Sampling

Age-matched (3–7 months old) adult male *C57BL/6 WT* and *SIRT3 KO (SIRT3^−^/^−^)* mice (*n* = 4 in each group) were obtained from Jackson Laboratory (*29-Sirt3^tm1.1Fwa^/J* strain; Bar Harbor, ME, USA). Mice were housed in a temperature-controlled environment (22 ± 1 °C) with a 12-h light/dark cycle and provided free access to standard chow and water. All experimental procedures were approved by the Institutional Animal Care and Use Committee (IACUC) of North Carolina Central University and conducted in accordance with NIH guidelines for the care and use of laboratory animals (Animal protocol number 11-29-2007).

Mice were anesthetized using carbon dioxide (CO_2_) and were transcardially perfused with cold phosphate-buffered saline (PBS) to remove circulating blood prior to tissue collection. Brain tissues were rapidly removed, flash-frozen in liquid nitrogen, and stored at −80 °C until further use.

### 2.2. Protein Extraction and Quantification

Frozen brain tissues, one hemisphere excluding cerebellum, were homogenized in an ice-cold RIPA lysis and extraction (Thermo Fisher Scientific, Waltham, MA, USA) buffer and a cocktail of protease and phosphatase inhibitor (Thermo Fisher Scientific). The homogenates were centrifuged at 14,000× *g* for 15 min at 4 °C, and the supernatants were collected for analysis. Protein concentration was determined using the bicinchoninic acid (BCA) assay (Thermo Fisher Scientific) according to the manufacturer’s instructions.

### 2.3. Western Blotting

An equal amount of protein lysates (30 μg) for each sample was separated on 4–12% Bis-Tris NuPAGE™ gels (Invitrogen, Carlsbad, CA, USA) and transferred to PVDF membranes (Millipore Sigma, Burlington, MA, USA) using an XCell SureLock mini-cell system (Invitrogen, Carlsbad, CA, USA). After transfer, membranes were blocked in a 1:1 solution of Li-COR Odyssey Blocking buffer (Li-COR Biosciences Inc., Linkoln, NE, USA) and PBS. The membranes were subsequently probed with an anti-SIRT3 antibody (1:1000, Rabbit Monoclonal Antibody Cat. #5490; Cell Signaling Technology, Boston, MA, USA), and an anti-beta actin antibody (cat. #MA5-15739; Invitrogen) as loading control, overnight in a cold room. Next morning, the blots were then incubated with secondary antibodies (IRDye 680RD Goat anti-Rabbit secondary antibodies, cat. #926-68071, and IRDye 800CW Goat anti-Mouse secondary antibodies Cat. #926-32210, LI-COR Biosciences Inc., Linkoln, NE, USA). The images were acquired using Li-COR Odyssey CLX Imaging System (Li-COR Biosciences Inc., Linkoln, NE, USA).

### 2.4. Antibody Microarray Analysis

The following protein microarray kits were purchased from R&D Systems Inc. (Minneapolis, MN, USA) and used in the present study: (1) Proteome Profiler Mouse Angiogenesis Array Kit (Catalog: ARY015), which detects 53 mouse angiogenesis-related proteins; (2) Proteome Profiler Mouse Apoptosis Array Kit (Catalog: ARY031), which measures 21 apoptosis-related proteins; (3) Proteome Profiler Mouse Chemokine Array Kit (Catalog: ARY020), which determines 28 mouse chemokine-related proteins; and (4) Proteome Profiler Mouse XL Cytokine Array Kit (Cat# ARY028), which spots 111 cytokines. An example of the protein array from the SIRT3 KO and WT brain samples is given in Supplemental Figure S1.

The microarray experiments were conducted according to the instructions from the manufacturers. In brief, the membranes were blocked for 1 h at room temperature with the blocking buffer included in the kits. Brain homogenates from 4 animals in each group were pooled, diluted to 300 µg/mL, mixed with a cocktail of biotinylated detection antibodies, and incubated with gentle agitation at 4 °C overnight with pre-blocked antibody membranes. After washing to remove unbound proteins, the membranes were incubated with a biotinylated detection antibody cocktail, followed by horseradish peroxidase (HRP)-conjugated streptavidin at room temperature on a rocking platform shaker for 30 min. The advantages of using pooled samples include reduced biological variability, ethical advantage for reducing the animal numbers, and cost efficiency. However, it obscures the inter-individual biological variability and reduces the statistical power for detecting subtle but biologically relevant differences. The analytics were visualized using the enhanced chemiluminescence (ECL) detection reagents provided in the kit, and the membranes were scanned using an Invitrogen iBright^TM^ FL1500 imaging system (Thermo Fisher Scientific). Densitometric analyses for angiogenic, apoptotic, chemokine, and cytokine membranes were performed using iBright Analysis Software. There are duplicate dot spots for each protein on each membrane, and the experiments were repeated once. Therefore, each value reflects the average of 4 measurements.

### 2.5. Data Normalization and Statistical Analysis

The chemiluminescent signals on membranes between the SIRT3 KO and WT groups were normalized by subtracting the mean negative control values on the membrane from the raw data and were followed by dividing the positive control mean value on the WT blots (reference array) by the positive control mean value on the SIRT3 KO blots. The signals from the SIRT3 KO group were compared with the corresponding spot signal obtained from the WT group. The calculation formula was as follows:

X(Ny) = X(y) * P1/P(y)

where 

P1 = the mean signal density of Positive Control spots on the reference array,

P(y) = the mean signal density of Positive Control spots on Array “y”,

X(y) = the mean signal density for spot “X” on the Array for sample “y”,

X(Ny) = the normalized signal intensity for spot “X” on Array “y”.

Statistical analyses were conducted using GraphPad Prism 10.0 (GraphPad Software, San Diego, CA, USA). The array optical densities for each protein marker on the WT membranes were converted to 1 and the array optical densities on SIRT3 KO membranes were expressed as a ratio to the WT group. A Mann–Whitney test was used for statistical analysis. Differences were considered to be statistically significant at *p* < 0.05.

## 3. Results

### 3.1. Confirmation of SIRT3 KO

**Figure 1:**
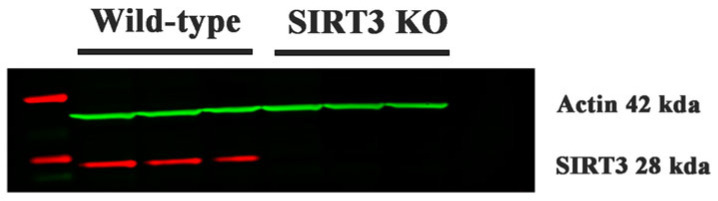
Confirmation of SIRT3 KO by Western blotting. Absence of SIRT3 protein bands are observed in SIRT3 KO brain samples. Red band, SIRT3 protein; green band, β-actin.

Western blotting was used to confirm SIRT3 protein expression in both the WT and SIRT3 KO mouse brain samples. As shown in Figure 1, while SIRT3 protein bands are clearly visible in the WT samples, they are absent in the SIRT3 KO samples, confirming the lack of SIRT3 protein expression in the knockout mice.

### 3.2. SIRT3 Deletion Alters the Expression of Angiogenic Proteins in Mouse Brain Tissue

**Figure 2:**
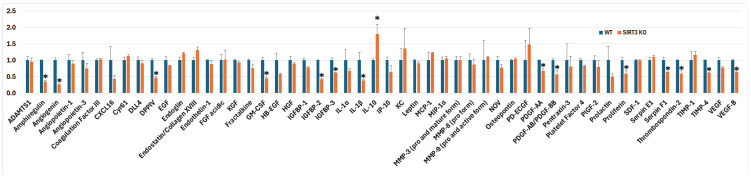
Fold change of the angiogenesis microarray. Deletion of SIRT3 led to suppression of the majority angiogenesis-related protein markers. Error bars are standard deviations; * *p* < 0.05.

As shown in Figure 2, among the 53 detected angiogenesis-related proteins, the SIRT3 KO mice displayed a distinct pattern of protein expression compared with the WT controls. Notably, 14 angiogenic factors, including amphiregulin, angiogenin, DPPIV, GM-CSF, IGFBP-2, IGFBP-3, IL-1β, PDGF-AA, PDGF-AB/PDGF-BB, proliferin, serpin F1, thrombospondin-2, TIMP-4, and VEGF-B were significantly decreased in the SIRT3 KO mice. By contrast, IL-10 had a significant increase in the SIRT3 KO mice. The remaining 39 angiogenic factors were not significantly changed in the SIRT3 KO compared to the WT samples. These results suggest that the deletion of SIRT3 had a suppressive effect on the angiogenesis processes.

### 3.3. Deletion of SIRT3 Increases Apoptotic Signaling in the Brain

**Figure 3:**
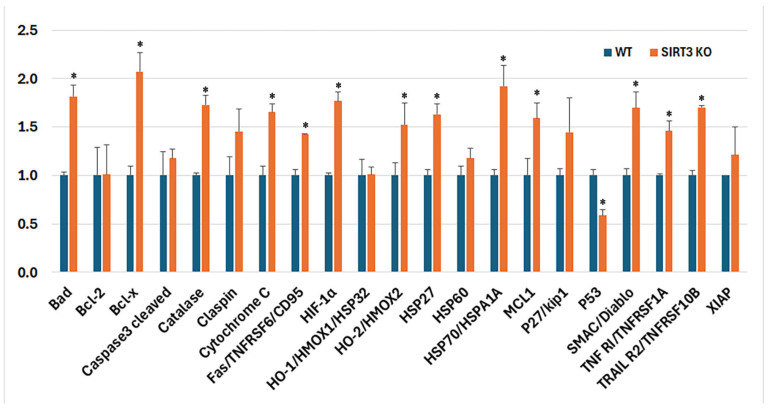
Fold change of apoptosis signaling array. SIRT3 KO mice exhibited a higher expression of both pro- and anti-apoptotic markers. Error bars are standard deviations; * *p* < 0.05.

To explore whether SIRT3 loss affects apoptotic pathways, proteins associated with apoptosis were compared between the two genotypes (Figure 3). The SIRT3 KO mice exhibited increased expression of pro-apoptotic markers including BAD, cytochrome c, Fas, HIF-1α, Smac/DIABLO, TNF R1/TNFRSF1A, and TRAIL R2. By contrast, the p53 level was significantly decreased. Additionally, anti-apoptotic and stress-related factors, including Bcl-x, catalase, HO-2/HMOX2, HSP27, HSP70/HSPA1A, and MCL1 were also increased in the SIRT3 KO mice. Overall, the deletion of SIRT3 enhanced the expression of both pro- and anti-apoptotic proteins.

### 3.4. SIRT3 Deletion Alters Chemokine Expression in Brain Tissue

**Figure 4:**
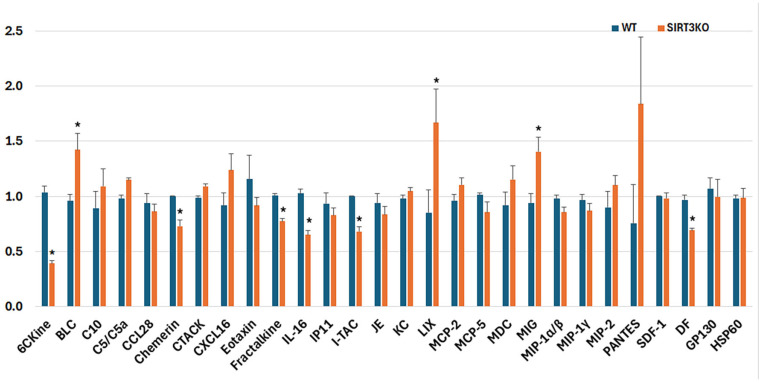
Fold change of the chemokine array. Deletion of SIRT3 leads to a suppression of 6CKine, chemerin, fractalkine, IL-16, I-TAC, and DF, and an elevation of BLC, LIX, and MIG. Error bars are standard deviations; * *p* < 0.05.

Chemokine profiling of brain homogenates revealed significant alterations in chemokine expressions associated with SIRT3 deletion. Of the 28 detected chemokines (Figure 4), 6 chemokines were significantly decreased (6CKine/CCL21, chemerin/CCRL2, fractalkine/CXCL1, IL-16, I-TAC/CXCL11, and Duffy antigen (DF)) in the SIRT3 KO compared with the WT brain samples. By contrast, three chemokines (BLC/CXCL13, LIX/CXCL5, and MIG/CXCL9) showed increased levels, while the remaining chemokines were not significantly altered in the SIRT3 KO mice relative to the WT group. These findings suggest an overall shift in the brain chemokine milieu following SIRT3 deletion, characterized by downregulation of specific homeostatic and immune-regulatory chemokines alongside upregulation of a subset of inflammatory chemokines. Notably, the decreased chemokines include molecules involved in leukocyte trafficking and neuroimmune surveillance (e.g., 6CKine), whereas the increased chemokines (e.g., BLC and MIG) are commonly associated with pro-inflammatory responses.

### 3.5. SIRT3 Deletion Selectively Affects Inflammatory Cytokines in Mouse Brain Tissue

**Figure 5:**
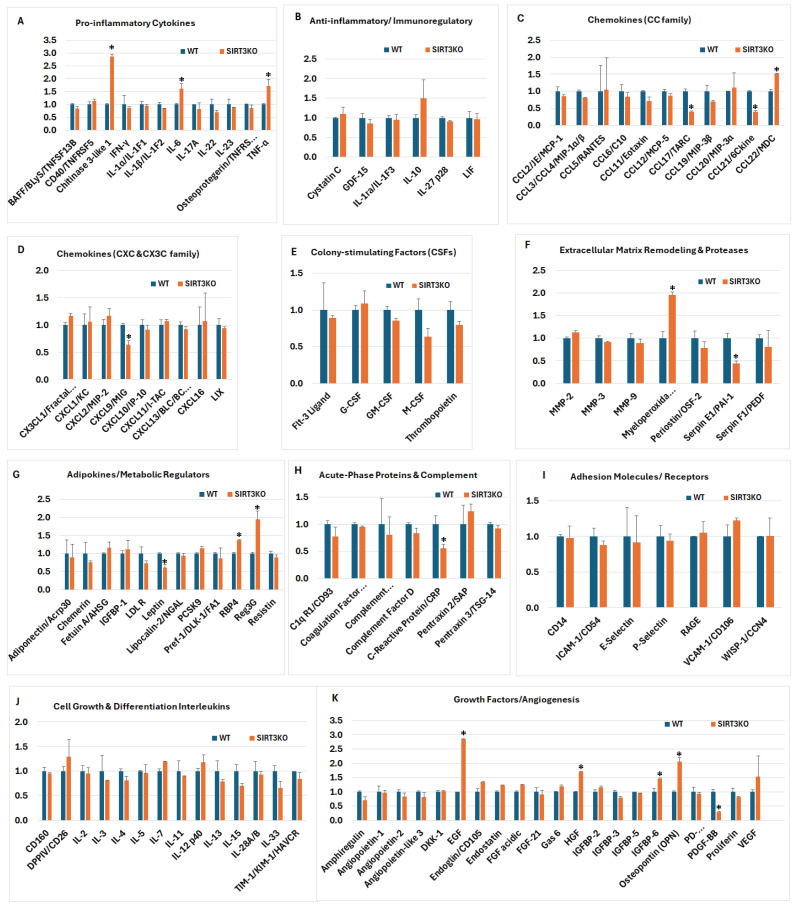
Fold changes of inflammatory cytokines. Deletion of SIRT3 results in selective and pathway-specific alterations, with 7 analytics decreased, 11 increased, and many signaling modules unchanged. (**A**) Pro-inflammatory cytokines; (**B**) Anti-inflammatory cytokines; (**C**) CC family chemokines; (**D**) CXC and CX3C family chemokines; (**E**) Colony-stimulating factors; (**F**) Extracellular matrix remodeling and proteases; (**G**) Adipokines and metabolic regulators; (**H**) Acute-phase proteins and complements; (**I**) Adehesion molecules and receptors; (**J**) Cell growth and differentiation interleukins; (**K**) Growth and angiogenesis factors. Error bars are standard deviations; * *p* < 0.05.

A total of 111 cytokines were analyzed using the Proteome Profiler Mouse XL Cytokine Array Kit. These cytokines were categorized into 11 subgroups, as shown in Figure 5, and their major functions are summarized in Supplemental Table S1. Overall, 7 cytokines in the SIRT3 KO brains (CCL17/TARC, CCL21/6Ckine, c-reactive protein, CXCL19/MIG, leptin, PDGF-BB, and serpin E1/PAI-1) significantly decreased compared with the WT controls. By contrast, 11 cytokines (chitinase 3-like 1, CCL22/MDC, EGF, HGF, IGFBP-6, IL-6, myeloperoxidase, osteopontin, RBP4, Reg3G, and TNF-α) were significantly increased in the SIRT3 KO mice. The fold changes (SIRT3 KO versus WT control mice) of the cytokines in each subgroup are shown in Figure 5A–K.

An analysis of the pro-inflammatory cytokines indicated that the loss of SIRT3 resulted in a selective, rather than global amplification of inflammatory mediators. Among the panel tested, chitinase 3-like 1, IL-6, and TNF-α were elevated dramatically in the SIRT3 KO samples (Figure 5A), whereas classical Th1/Th17 cytokines (IFN-γ, IL-17A, IL-22, IL-23), IL-1 family members, and IL-6 remained largely unchanged. Other factors, such as cystatin C, GDF-15, IL-1ra/IL-1F3, IL-27 p28, and LIF, exhibited no significant alterations (Figure 5B).

An evaluation of the CC family and CXCL chemokines demonstrated that SIRT3 deficiency did not broadly affect chemokine production but instead led to the selective suppression of specific immune-regulatory chemokines, notably CCL17/TARC, CCL21/6Ckine, and CXCL9/MIG. Most classical inflammatory chemokines, including CCL2/MCP-1, CCL5/RANTES, CXCL1/KC, CXCL2/MIP-2, CXCL10/IP-10, CXCL11/ITAC, CXCL13, CXCL16, CX3CL1, and LIX remained comparable between the genotypes (Figure 5C,D).

An assessment of colony-stimulating factors (CSFs) revealed a selective effect of SIRT3 deletion on hematopoietic growth factor expression. While Flt-3 ligand, G-CSF, and GM-CSF levels remained comparable between the WT and SIRT3 KO samples. M-CSF and thrombopoietin modestly decreased in the absence of SIRT3 (Figure 5E), but these changes did not reach statistical significance.

An analysis of extracellular matrix remodeling and protease regulators revealed that SIRT3 deficiency induced a selective remodeling phenotype. Among the factors examined, myeloperoxidase was significantly increased in the SIRT3 KO samples, while serpin E1/PAI-1 significantly reduced. By contrast, expression of major matrix metalloproteinases (MMP-2, MMP-3, MMP-9) and other protease-related factors, including periostin/OSF-2 and serpin F1/PEDF, remained close to the WT levels (Figure 5F).

An investigation of adipokines and metabolic regulators demonstrated that SIRT3 deficiency did not broadly disrupt systemic metabolic signaling, but instead induced a highly selective change; most notably, a significant upregulation of RBP4 and Reg3G, and a downregulation of leptin. Other classical adipokines, including adiponectin, chemerin, and resistin, as well as lipid-related regulators, such as PCSK9 and LDL-R, remained near the WT levels (Figure 5G).

Relative expressions of acute-phase proteins and complement components revealed that SIRT3 deficiency did not elicit a generalized acute-phase inflammatory response. Most measured factors, including complement-related proteins, coagulation-associated factors, and pentraxins (pentraxin 2/SAP and pentraxin 3/PTX3), remained comparable between the SIRT3 KO and WT samples. By contrast, C-reactive protein (CRP) was significantly reduced in the SIRT3 KO conditions (Figure 5H).

An analysis of adhesion molecules and immune receptors revealed no major differences between the SIRT3 KO and WT samples. Core mediators of leukocyte adhesion and endothelial activation, including ICAM-1, VCAM-1, e-selectin, and p-selectin, stayed at levels comparable to the WT controls. Similarly, expressions of CD14, RAGE, and WISP-1/CCN4 were largely unchanged (Figure 5I).

An evaluation of the interleukins involved in cell growth and differentiation demonstrated that SIRT3 deletion did not broadly alter the mediators governing immune cell proliferation, survival, or lineage commitment. The expression levels of key T cell growth factors (IL-2, IL-7, IL-15), Th1/Th2 differentiation signals (IL-4, IL-5, IL-13), and innate–adaptive bridging cytokines (IL-12p40, IL-33) persisted at comparable levels between the SIRT3 KO and WT samples. Similarly, expressions of immune regulatory receptors and cofactors, including CD26/DPPIV, CD160, and TIM-1/HAVCR, were largely unchanged (Figure 5J).

An analysis of the growth factors and angiogenesis-associated mediators demonstrated that SIRT3 deletion induced a selective remodeling of pro-growth and pro-angiogenic signaling. Notably, the SIRT3 KO samples exhibited significant upregulation of EGF, HGF, IGFBP-6 and osteopontin, and downregulation of PDGF-BB (Figure 5K).

In summary, our comprehensive proteomic profiling revealed that SIRT3 deletion profoundly disrupts angiogenic, apoptotic, and neuroimmune signaling in the mouse brain. Loss of SIRT3 markedly suppressed multiple angiogenic factors while simultaneously activating intrinsic and extrinsic apoptotic pathways, consistent with impaired cerebrovascular support and heightened neuronal stress. Chemokine and cytokine analyses uncovered the selective reprogramming of immune signaling, characterized by a reduced homeostatic immune surveillance and an increased expression of inflammatory mediators linked to glial activation. Notably, elevated IL-6, TNF-α, and chitinase 3-like 1 are molecules strongly associated with neurodegenerative pathology, suggesting a shift toward a chronic, injury-prone inflammatory milieu. Together, these findings indicate that SIRT3 loss creates a proteomic signature compatible with the mechanisms driving progressive neurodegeneration.

## 4. Discussion

The present study demonstrates that the deletion of SIRT3 leads to declined levels of the majority of the proteins involved in angiogenesis, increased levels of apoptosis protein markers, and mixed responses among the detected chemokines and cytokines in brain tissues. These findings provide new insights into the immune regulatory functions of SIRT3.

Angiogenesis is the process of new blood vessel formation from the existing blood vessels. It starts with degradation of the blood vessel basement membrane, blood endothelial cell proliferation and migration, and the formation of endothelial sprout. Then, capillary tubes develop with a tight junction between the endothelial cells, and the deposition of new basement membrane. Mechanistically, SIRT3 influences endothelial cell metabolism and redox balance in ways that favor angiogenic signaling. For example, endothelial-specific SIRT3 deletion reduces glycolytic flux, lowers VEGF and Angiopoietin-1 expression, and impairs hypoxia-induced neovascularization, suggesting that SIRT3 supports the metabolic demands of angiogenic endothelial cells [26,27]. Moreover, SIRT3 reduces mitochondrial reactive oxygen species and stabilizes factors such as HIF-1α that enhance pro-angiogenic gene transcription under hypoxia, while SIRT3 deficiency is associated with microvascular rarefaction and diminished vessel density in multiple pathological settings [28,29]. Our results demonstrate that loss of SIRT3 significantly suppressed angiogenesis-related factors, which supports the role of SIRT3 in vascular remodeling and endothelial cell function under physiological conditions. Previous studies have shown that SIRT3 promotes endothelial cell survival and angiogenesis by enhancing mitochondrial ATP production [7,26,30,31]. The reduction in angiogenic proteins observed here suggests that SIRT3 deficiency disrupts pro-angiogenesis signaling, thereby hindering vascular repair capacity.

Interleukin-10 (IL-10) suppresses pro-inflammatory cytokine production and promotes tissue repair [32]. SIRT3 and IL-10 reinforce each other [33]. The increased IL-10 levels suggest that either SIRT3 has a differential action on different angiogenic factors, or a reactive response to enhanced inflammation in the SIRT3 KO animals. The latter is supported by the findings that deletion of SIRT3 activated pro-apoptotic factors and elevated several pro-inflammatory proteins, as shown in Figure 2 and Figure 5.

Apoptosis is a programmed cell death triggered by various insults that cause the release of pro-apoptotic factors and the activation of downstream proteases. SIRT3 critically regulates apoptosis by modulating oxidative stress, mitochondrial integrity, and energy metabolism [34,35,36]. Our results demonstrate that the deletion of SIRT3 increases the levels of BAD, cytochrome c, and Smac/DIABLO, suggesting that SIRT3 oblation causes cell stress (reflected by increases in HSP27 and HSP70) that may activate the intrinsic (mitochondria-mediated) cell death pathway. In the meantime, Fas, TNF R1, and TRAIL-R2 were also increased in the SIRT3 KO mice, suggesting activation of the extrinsic (receptor-mediated) cell death pathway. The activation of cell death pathways may cause the body to initiate counter reactions reflected by the activation of cell survival signals. This proved to be the case, since anti-apoptotic Bcl-x, Mcl-1, and catalase significantly increased in the SIRT3 KO mice. The increases of HIF-1α and HO-2 suggest that the deletion of SIRT3 may affect cell metabolisms and redox/hypoxia adaption. It is likely that chronic metabolic stress favored p53 suppression, which implies a divergence from canonical DNA damage and mediated apoptosis, favoring survival under prolonged metabolic stress. Our results are consistent with the literature reports showing that deletion of the SIRT3 gene causes neuronal cell death in animal models of traumatic brain injury, cerebral ischemic stroke, epilepsy, and Parkinson’s disease through modulating oxidative stress, HIF-1α, VEGF, and mitochondrial functional integrity [31,37].

Collectively, this profile is consistent with an apoptosis-primed but stress-adapted neuronal state, which may contribute to progressive neurodegeneration under sustained SIRT3 loss.

Our chemokine profiling demonstrates that SIRT3 deletion profoundly alters chemokine expression in the mouse brain, with both reductions and increases in discrete chemokines. These results expand on the growing literature implicating SIRT3 as a regulator of neuroinflammation and immune signaling in vivo. The observed reduction in chemokines, such as 6CKine/CCL21, chemerin, and fractalkine/CX3CL1, suggests that homeostatic immune surveillance and neuron–glia signaling pathways may be suppressed in the absence of SIRT3. 6CKine/CCL21 plays a role in directing leukocyte migration and maintaining central immune vigilance; its decrease may reflect impaired recruitment or retention of beneficial immune cells [38,39]. Fractalkine/CX3CL1 is primarily expressed by neurons and signals through microglial CX3CR1 to regulate microglial activation and neuroprotective interactions of inflammation [40]. Reduced fractalkine might therefore represent a loss of neuron-to-microglia regulatory signaling, potentially predisposing to be dysregulated. The upregulation of BLC/CXCL13, LIX/CXCL5, and MIG/CXCL9 in the SIRT3 KO mouse brain suggests a shift toward a pro-inflammatory or immune-attractant profile. CXCL9 and CXCL13 are typically associated with T-cell and B-cell recruitment in inflammatory contexts [41,42]; their elevation may reflect compensatory immune signaling in response to neuronal stress or ongoing microglial activation in SIRT3 deficient mice. Elevated expressions of such chemokines have been linked with neuroinflammatory conditions and may contribute to secondary injury processes.

The altered chemokine signature complements the existing evidence that SIRT3 deficiency enhances inflammatory responses and modulates innate immunity in the brain. SIRT3 has been shown to limit activation of key inflammatory pathways, including the NLRP3 inflammasome, and to reduce pro-inflammatory cytokines following hypoxic–ischemic injury [43]. Moreover, recent studies have demonstrated that SIRT3 suppresses activation of the DNA-sensing pathways, such as the cGAS–STING pathway in astrocytes, thereby attenuating neuroinflammation after cerebral ischemia [44]. Our chemokine profiling extends this framework by indicating that SIRT3 deletion not only affects classical cytokine pathways but reshapes the chemokine networks involved in immune cell recruitment, glia activation, and possibly blood–brain barrier dynamics, which are critical in neuroimmune homeostasis and injury responses.

Inflammatory cytokines function as signaling molecules in regulating the inflammatory processes that stimulate the immune system to initiate tissue repair. However, a sustained inflammatory response may amplify the tissue damage processes by producing excessive toxic factors. Our multiplex profiling of inflammatory cytokines reveals that SIRT3 deletion induces coordinated alterations across the inflammatory, redox, metabolic, and angiogenic pathways that are highly relevant to neurodegeneration. Collectively, these changes position SIRT3 as a central mitochondrial rheostat integrating immunometabolism and vascular signaling. In the CNS, where mitochondrial integrity critically governs neuronal survival, synaptic plasticity, and glial reactivity, disruption of SIRT3-dependent deacetylation programs is expected to shift the balance toward a pro-inflammatory and pro-degenerative state [34,45]. We will summarize the discussion in the following categories: inflammatory signaling cascades, redox dysregulation and protease remodeling, metabolic reprogramming and adipokine signaling, and growth factor signaling.

*Inflammatory signaling cascades*. Figure 5A–D demonstrate the selective amplification of pro-inflammatory cytokines and chemokines in the SIRT3 KO samples, including increased chitinase 3-like 1 (CHI3L1), IL-6, TNF-α, and chemokine CCL22, alongside the relative preservation or reduction of other regulatory mediators. These changes map directly onto canonical NF-κB and JAK/STAT pathways. SIRT3 normally suppresses mitochondrial ROS production through deacetylation of SOD2 and regulation of electron transport enzymes [25,46]. In its absence, enhanced mitochondrial ROS can activate inhibitor of nuclear factor-κB (IκB) kinase (IKK), thereby promoting NF-κB nuclear translocation and transcription of IL-6, TNF-α, and multiple chemokines [47]. CHI3L1 has been categorized both as a pro- and anti-inflammatory cytokine. In clinic settings, it has been used as an early inflammation marker. Elevated levels of CHI3L1 are associated with several diseases, including asthma, arthritis, sepsis, diabetes, liver fibrosis, and coronary artery disease [48]. The elevation of IL-6 and related mediators further propagates STAT3 signaling, which in neurodegenerative settings drives reactive gliosis and sustains inflammatory amplification loops [49]. These results agree with a previous report showing that overexpression of SIRT3 in the CA1 region suppressed the levels of TNF-α, IL-1β, and IL-6 [50]. Decreased levels of the chemoattractant CXCL9 is in line with augmented IFN-γ- and STAT1-dependent immune recruitment pathways, which are prominent in neurodegenerative diseases [51]. Thus, SIRT3 loss appears to shift the neuroimmune microenvironment toward a feed-forward NF-κB/STAT axis characterized by heightened innate activation and adaptive immune engagement—two features strongly associated with progressive neuronal dysfunction [52].

*Redox dysregulation and protease remodeling.* Figure 5F reveals the marked upregulation of myeloperoxidase (MPO), alongside a change in serpin E1 in the SIRT3 KO condition. SIRT3 deficiency is well established to increase mitochondrial oxidative stress via hyperacetylation and inactivation of SOD2 [25,46]. Elevated MPO further amplifies oxidative damage by generating hypochlorous acid and related reactive intermediates. It promotes tissue damage in chronic disease by generating free radicals [53]. Yan et al. reported that SIRT3 gene deletion increases the level of MPO in the lungs after sepsis infection [54], which agrees with our results. In neurodegeneration, MPO-derived oxidants exacerbate protein misfolding and blood–brain barrier (BBB) compromise [55]. The increased levels of MPO in the SIRT3 KO mice made the animals vulnerable to inflammatory stimuli and disease development. Serpin E1 is a protease inhibitor expressed highly in the nervous system [56,57]. Suppression of serpin E1 may lead to enhanced neurotoxicity, neuroinflammation, microglial activation, oxidative stress, and BBB disruption [58,59]. This is at variance with the literature reports showing that loss of SIRT3 increases the level of serpin E1 in animal lung tissue [60]. It is possible that the effect of SIRT3 deletion on serpin E1 is tissue dependent.

*Metabolic reprogramming and adipokine signaling.* Figure 5G indicates alterations in adipokines and metabolic regulators (leptin, RBP4, Reg3G). Leptin is a hormone that regulates appetite, food intake, and body metabolism [61]. RBP4 (retinol binding protein 4) is a lipocalin family protein. Its primary function is to transport retinol (vitamin A) in the blood from the liver to peripheral tissues. Overexpression of RBP4 causes progressive retinal degeneration [62]. Reg3G directly targets certain bacteria in the gastrointestinal tract and functions as a hormone regulating glucose metabolism and energy balance [63]. SIRT3 is a master regulator of mitochondrial fatty acid oxidation and TCA cycle enzyme activity [64]. Its deletion promotes metabolic inflexibility and increases glycolytic reliance, a shift often accompanied by inflammatory activation in microglia and astrocytes. Metabolic reprogramming toward glycolysis is characteristic of reactive glial states and is tightly linked to IL-1β and IL-6 production [65]. Therefore, SIRT3 deletion may rewire metabolic processes and enforce inflammatory cascades. As one of the major functions of SIRT3 is to regulate energy metabolism, it is not surprising that deletion of SIRT3 affects the metabolic regulators.

*Growth factor signaling*. Figure 5K demonstrates the robust induction of growth factors, including EGF, HGF, IGFBP-6, and osteopontin (OPN). SIRT3 negatively regulates HIF-1α stabilization under normoxic conditions by limiting mitochondrial ROS accumulation [27]. Loss of SIRT3 may therefore enhance ROS-dependent HIF-1α signaling, as confirmed in Figure 3, leading to compensatory growth factor responses. Elevated osteopontin, a multifunctional cytokine linked to microglial activation, has been associated with Alzheimer’s disease [66,67]. One should be aware that the SIRT3 KO mice used in this study are nicotinamide nucleotide transhydrogenase (NNT) deficient. NNT regulates mitochondrial NADPH production and redox balance, thereby sustaining glutathione and thioredoxin antioxidant systems [68]. Some of the observed alterations in the SIRT3 KO mice may reflect the additive or synergistic effects of combined SIRT3 and NNT deficiency rather than SIRT3 loss alone.

## 5. Conclusions

This study identifies SIRT3 as a critical mitochondrial regulator of angiogenesis, cell survival, and neuro-immune homeostasis in the brain. SIRT3 deficiency suppresses vascular remodeling, primes apoptotic signaling, and selectively amplifies the inflammatory pathways implicated in neurodegenerative disease progression. The coordinated increase in oxidative stress markers, pro-apoptotic proteins, and neurodegeneration-associated cytokines suggests that the loss of SIRT3 promotes a brain environment vulnerable to chronic neuronal dysfunction and degeneration. Rather than inducing global inflammation, SIRT3 deletion drives the targeted immuno-metabolic dysregulation that mirrors the pathogenic features observed in disorders such as Alzheimer’s disease, Parkinson’s disease, and ischemic brain injury. These findings highlight SIRT3 as a potential therapeutic target for mitigating neurodegeneration by preserving mitochondrial integrity, vascular support, and immune balance.

## Data Availability

The data that support the findings of this study are available upon reasonable request from the corresponding author. The data are not publicly available due to ethical considerations.

## Supplementary Materials

The following supporting information can be downloaded at: https://www.mdpi.com/article/10.3390/brainsci16030270/s1, Figure S1. Sample of microarray. Table S1. Classifications and functions of the 111 detected cytokines.
