# Supplementary material for: Comprehensive Proteomic Profiling Reveals Dysregulation of Angiogenesis and Inflammatory Pathways in the Brains of SIRT3 Knockout Mice
Source: Brain Sci. 2026 Feb 28;16(3):270. doi: 10.3390/brainsci16030270 (PMC13024698; doi:10.3390/brainsci16030270)

brain sciences

MDPI

Supplemental data

Figure S1. Example of microarray. Proteins affected by SIRT3 deletion are outlined on the membranes and their names are listed on right side of the membranes.

Apoptosis Array

1	2	3	4	5	6	7	8
Ref	Ref	Bad	Bad	Bcl-2	Bcl-2	Ref	Ref
		Bcl-x	Bcl-x	Caspase 3(cleaved)	Caspase 3(cleaved)		
Catalase	Catalase	Claspin	Claspin	Cytochrome C	Cytochrome C	Fas/TNFRSF6/ CD95	Fas/TNFRSF6/ CD96
HIF-1α	HIF-1α	H0-1/HMOX1/ HSP32	H0-1/HMOX1/ HSP32	H0-2/HMOX2	H0-2/HMOX2	HSP27	HSP27
HSP60	HSP60	HSP70/HSPA1A	HSP70/HSPA1A	MCL-1	MCL-1	p27/Kip1	p27/Kip1
P53	P53	SMAC/Diablo	SMAC/Diablo	TNF RI/TNFRSF1A	TNF RI/TNFRSF1A	TRAIL R2/ TNFRSF10B	TRAIL R2/ TNFRSF10B
Ref	Ref			XIAP	XIAP	Negative Control	Negative Control

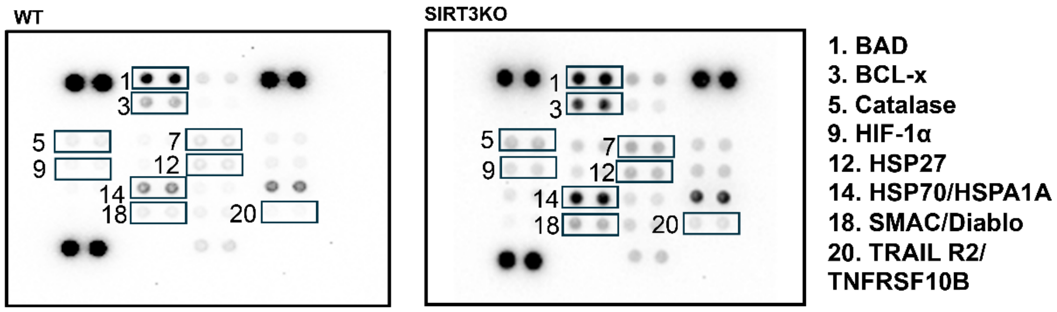

Supplement: Supplementary file 1 [file brainsci-16-00270-s001.zip › brainsci-4063978-Figure S1.pdf]
