# Supplementary material for: Comprehensive Proteomic Profiling Reveals Dysregulation of Angiogenesis and Inflammatory Pathways in the Brains of SIRT3 Knockout Mice
Source: Brain Sci. 2026 Feb 28;16(3):270. doi: 10.3390/brainsci16030270 (PMC13024698; doi:10.3390/brainsci16030270)
Supplement: Supplementary file 1 [file brainsci-16-00270-s001.zip › brainsci-4063978-Table SI.pdf]

Supplementary data

Table 1. Classifications and functions of the 111 detected cytokines

Pro-inflammatory Cytokines

BAFF/BLyS/TNFSF13B	Regulates B-cell survival, maturation, and antibody production. Proinflammation
CD40/TNFRSF5	Co-stimulatory receptor mediating B-cell and macrophage activation. Proinflammation
Chitinase 3-like 1	Pro- and anti-inflammation. Glycoprotein linked to tissue remodeling and inflammation.
IFN-γ	Activates macrophages and promotes Th1 immune responses; key mediator of inflammation.
IL-1α/IL-1F1	Induces fever and inflammatory gene expression; promotes leukocyte recruitment.
IL-1β/IL-1F2	Potent mediator of inflammation and fever; stimulates cytokine and adhesion molecule expression.
IL-6	Drives acute-phase response, B cell maturation, and chronic inflammation.
IL-17A	Stimulates neutrophil recruitment and pro-inflammatory cytokine release.
IL-22	Promotes epithelial barrier repair and antimicrobial peptide production.
IL-23	Supports Th17 cell maintenance and inflammation in autoimmune disorders.
Osteoprotegerin /TNFRSF11B	Decoy receptor inhibiting RANKL; regulates bone turnover and vascular calcification. Regulati
TNF-α	Central mediator of inflammation; induces apoptosis, cytokine release, and vascular adhesion.

Anti-inflammatory Cytokines

Cystatin C	Cysteine protease inhibitor; marker of kidney function and inflammation.
GDF-15	Stress-induced cytokine regulating inflammation and metabolism.
IL-1ra/IL-1F3	Natural antagonist of IL-1 receptor; limits IL-1-driven inflammation.
IL-10	Suppresses macrophage activation and pro-inflammatory cytokine production.
IL-27 p28	Regulates Th1/Th17 balance and suppresses excessive inflammation.
LIF	Induces cell differentiation, survival, and anti-apoptotic signaling.

Chemokines (CC family)

CCL2/JE/MCP-1	Recruits monocytes and memory T cells to inflammatory sites.
CCL3/CCL4/MIP-1α/β	Attracts macrophages, NK, and T cells; enhances inflammation.
CCL5/RANTES	Recruits T cells, eosinophils, and basophils; supports chronic inflammation.
CCL6/C10	Chemoattractant for monocytes and eosinophils; regulates immune responses.
CCL11/Eotaxin	Recruits eosinophils; key in allergic and asthmatic inflammation.
CCL12/MCP-5	Monocyte chemoattractant involved in inflammatory infiltration.
CCL17/TARC	Attracts Th2 lymphocytes; contributes to allergic inflammation.
CCL19/MIP-3β	Guides T and dendritic cells to lymphoid tissues.
CCL20/MIP-3α	Attracts immature dendritic cells and Th17 cells.
CCL21/6Ckine	Directs T cells and dendritic cells to lymphoid organs.
CCL22/MDC	Recruits Th2 cells and regulatory T cells to inflamed tissues.

Chemokine (CXC, CX3C Family)

CX3CL1/Fractalkine	Dual adhesion and chemotactic molecule for monocytes and T cells.
CXCL1/KC	Potent neutrophil chemoattractant; promotes inflammation.
CXCL2/MIP-2	Recruits neutrophils during acute inflammation.
CXCL9/MIG	Attracts activated T cells; induced by IFN-γ.
CXCL10/IP-10	Recruits Th1 cells and inhibits angiogenesis.
CXCL11/I-TAC	Strong chemoattractant for activated T cells; IFN-inducible.
CXCL13/BLC/BCA-1	Promotes B cell migration to follicles.
CXCL16	Recruits T cells and promotes scavenger receptor activity.
LIX	Neutrophil chemoattractant involved in tissue injury.

Colony-Stimulating Cytokines

Flt-3 Ligand	Stimulates differentiation of hematopoietic progenitors, especially dendritic and B cells.
G-CSF	Promotes neutrophil proliferation, differentiation, and mobilization from bone marrow.
GM-CSF	Stimulates granulocyte, macrophage, and dendritic cell production and activation.
M-CSF	Induces monocyte and macrophage survival and differentiation.
Thrombopoietin	Regulates platelet production and megakaryocyte development.

Growth Factors & Angiogenesis Regulators

Amphiregulin	EGFR ligand promoting epithelial proliferation and wound healing.
Angiopoietin-1	Promotes vascular stabilization and endothelial survival.
Angiopoietin-2	Antagonizes Ang-1; facilitates vascular remodeling and inflammation.
Angiopoietin-like 3	Regulates lipid metabolism and endothelial angiogenesis.

DKK-1	Wnt pathway inhibitor controlling bone formation and vascular calcification.
EGF	Stimulates cell proliferation and differentiation via EGFR signaling.
Endoglin/CD105	Co-receptor for TGF-β; regulates angiogenesis and endothelial function.
Endostatin	Inhibits angiogenesis and tumor vascularization.
FGF acidic (FGF-1)	Promotes fibroblast proliferation and angiogenesis.
FGF-21	Metabolic regulator with protective vascular and anti-inflammatory effects.
Gas 6	Activates TAM receptors (Axl, Mer, Tyro3); promotes cell survival and phagocytosis.
HGF	Stimulates endothelial motility, proliferation, and tissue regeneration.
IGFBP-2	Modulates IGF signaling and cell growth; metabolic regulation.
IGFBP-3	Major IGF carrier; controls IGF bioactivity and apoptosis.
IGFBP-5	Promotes cell differentiation and matrix formation.
IGFBP-6	Preferentially binds IGF-II; modulates growth and metabolism.
Osteopontin (OPN)	Adhesion molecule involved in bone remodeling and inflammation.
PD-ECGF/Thymidine phosphorylase	Pro-angiogenic enzyme involved in endothelial migration.
PDGF-BB	Promotes fibroblast proliferation and vessel maturation.
Proliferin	A Key regulator promoting angiogenesis
VEGF	Key endothelial mitogen driving angiogenesis and vascular permeability.

Adipokine and Metabolic Regulators

Adiponectin/Acrp30	Enhances insulin sensitivity; anti-inflammatory and anti-atherogenic.

Chemerin	Regulates adipocyte differentiation and immune cell chemotaxis.
Fetuin A/AHSG	Inhibits insulin signaling and modulates inflammation.
IGFBP-1	Regulates IGF availability; linked to glucose metabolism.
LDL R	Receptor for LDL; involved in lipid uptake and metabolism.
Leptin	Regulates appetite, metabolism, and immune activation.
Lipocalin-2/NGAL	Links metabolism and inflammation; iron transport and stress response.
PCSK9	Regulates LDL receptor degradation; controls cholesterol metabolism.
Pref-1/DLK-1/FA1	Inhibits adipocyte differentiation; regulates endocrine metabolism.
RBP4	Retinol transporter; associated with insulin resistance.
Reg3G	Antimicrobial lectin protecting mucosal surfaces.
Resistin	Modulates insulin sensitivity and promotes inflammation.

Acute-Phase Proteins & Complements

C1q R1/CD93	Complement receptor promoting phagocytosis and inflammation.
Coagulation Factor III/Tissue Factor	Initiates coagulation cascade; links thrombosis and inflammation.
Complement Component C5/C5a	Potent anaphylatoxin; recruits leukocytes and promotes inflammation.
Complement Factor D	Catalyzes activation of alternative complement pathway.
C-Reactive Protein (CRP)	Acute-phase protein enhancing complement activation and opsonization.
Pentraxin 2/SAP	Modulates innate immunity and fibrosis resolution.
Pentraxin 3/TSG-14	Local acute-phase protein regulating complement and inflammation.

Adhesion Molecules & Receptors

CD14	Co-receptor for LPS recognition; innate immune activation.
ICAM-1/CD54	Promotes firm adhesion of leukocytes to endothelium.
E-Selectin/CD62E	Mediates leukocyte rolling on endothelium during inflammation.
P-Selectin/CD62P	Mediates platelet and endothelial adhesion to leukocytes.
RAGE	Receptor for advanced glycation end-products; promotes inflammation and oxidative stress.
VCAM-1/CD106	Facilitates lymphocyte adhesion and transmigration.
WISP-1/CCN4	Regulates extracellular matrix formation and angiogenic signaling.

T/B-Cell Growth & Differentiation Interleukins

CD160	Immune checkpoint molecule regulating T-cell cytotoxicity.
DPPIV	Regulates body metabolism, inhibits glucagon-like peptide-1 (GLP-1)
IL-2	Stimulates T-cell proliferation and NK-cell activation.
IL-3	Supports proliferation of hematopoietic progenitor cells.
IL-4	Inhibits pro-inflammatory cytokine synthesis and promotes Th2 differentiation.
IL-5	Promotes eosinophil differentiation and survival.
IL-7	Critical for T and B lymphocyte development and homeostasis.
IL-11	Reduces inflammatory signaling; promotes tissue protection and hematopoiesis.
IL-12 p40	Component of IL-12 and IL-23; drives Th1 differentiation.
IL-13	Mediates anti-inflammatory and allergic Th2-type immune responses.
IL-15	Promotes NK-cell and memory $CD8^+$ T-cell survival.

IL-28A/B	Type III interferons; induce antiviral responses.
IL-33	Induces type 2 immune responses and tissue repair; modulates inflammation.
TIM-1/KIM-1/HAVCR	Immune and renal injury marker; mediates cell adhesion and phagocytosis.

Extracellular Matrix Remodeling & Proteases

MMP-2	Degrades type IV collagen; facilitates matrix remodeling and angiogenesis.
MMP-3	Breaks down extracellular matrix during tissue repair.
MMP-9	Degrades basement membrane components; promotes leukocyte migration.
Myeloperoxidase	Generates hypochlorous acid; contributes to oxidative tissue injury.
Periostin/OSF-2	ECM protein supporting tissue repair and fibrosis.
Serpin E1/PAI-1	Inhibitor of plasminogen activators; regulates fibrinolysis.
Serpin F1/PEDF	Anti-angiogenic and neuroprotective serpin family protein.
